# Supplementary material for: Low grade intravascular hemolysis associates with peripheral nerve injury in type 2 diabetes
Source: PLoS One. 2022 Oct 17;17(10):e0275337. doi: 10.1371/journal.pone.0275337 (PMC9576093; doi:10.1371/journal.pone.0275337)
Supplement: S1 File — Reagents and assays, T2D cohort ‘Diabelyse’, Inclusion parameters, Non-inclusion parameters, Blood sample collection, Flow cytometry for extracellular vesicles. (DOCX) [file pone.0275337.s001.docx]

**SUPPORTING INFORMATION**

**Low Grade Intravascular Hemolysis Associates with Peripheral Nerve Injury**

**in Type 2 Diabetes**

Sylvain Le Jeune, MD ^1,2^ ; Sihem Sadoudi, PhD ^1^ ; Dominique Charue, MSc ^1^ ; Salwa Abid, MSC ^1^ ; Jean-Michel Guigner, PhD ^3^ ; Dominique Helley, MD PhD ^1,4^ ; Hélène Bihan, MD PhD ^5^ ; Camille Baudry, MD ^6^ ; Hélène Lelong, MD PhD ^7^ ; Tristan Mirault, MD PhD ^1,8^ ; Eric Vicaut, MD PhD ^1,9^ ; Robin Dhote, MD PhD ^2^ ; Jean-Jacques Mourad, MD PhD ^10^ ; Chantal M. Boulanger, PhD ^1^; Olivier P. Blanc-Brude, PhD ^1^.

**Short title:**

Intravascular Hemolysis is a component of type 2 diabetes associated with peripheral neuropathy.

**Key Words:**

Type 2 diabetes, Intravascular hemolysis, Red blood cells, Hemoglobin, Heme, Extracellular vesicles, Peripheral Neuropathy.

**Manuscript data:** Le Jeune/2022/Version 1

**Correspondence:**

Olivier Blanc-Brude

Paris Center for Cardiovascular Research - Inserm U970

Hôpital Européen Georges Pompidou,

56 rue Leblanc, F-75015 PARIS, France

Tel : +33 / 1 53 98 80 61

e-mail : [olivier.blanc-brude@inserm.fr](mailto:olivier.blanc-brude@inserm.fr)

_____________

**Supplementary Materials & Methods**

**Reagents and assays**

Anti-Human CD235a-APC antibody (clone GA-R2 (HIR2)) and mouse IgG2b,κ-APC were obtained from BD Biosciences (BD Pharmingen, Le Pont-de-Claix, France; reference numbers 561775 and 555745). Human Annexin-A5-FITC was from Roche Diagnostics (Merck France, reference number 11858777001).

**T2D cohort ‘Diabelyse’**

‘Diabelyse’ was a biological collection registered with the national French ministry of health (n°DC-2011-1480). We included 109 adult T2D patients in the diabetology unit of Avicenne Hospital. Demographic, clinical, laboratory and treatment data were collected, including cardiovascular risk factors, micro- and macrovascular complications (Supplementary Table 1). 65 non-diabetic control volunteers were enrolled in Centre d’Examens de Santé (Bobigny) and Vascular Medicine Units of Avicenne, Hôtel-Dieu and European Georges Pompidou Hospitals between 18/01/2012 and 29/11/2016.

Eligibility criteria were carefully recorded in case report forms. Case ascertainment was made based on clinical and biological data, before sample anonymization. We performed a case-control study nested in the biological collection, basing the selection of patients (cases and controls) on the existence of biological samples and on patient records for the relevant clinical and biological data. We obtained ethical approval from committee CPP-IDF-VII on 01/02/2012. Written consent was obtained for all participants. Authors had no access to information that could identify individual participants during or after data collection.

Study size number could not be precisely defined as no data was available to formulate a hypothesis for the primary objective: No measurements of plasma absorbance and no measurements of intravascular hemolysis in T2 diabetic patients. Such biomarkers investigated in genetic blood disorders are difficult to transpose to T2 diabetes, where subclinical hemolysis was expected. Sensitivity analyses were not performed in this study, as missing data were not imputed.

The non-diabetic group included subjects with cardiovascular risk factors, mainly hypertension (HTN) (56%) and dyslipidemia (34%), in order to better illustrate the relative effect of T2D. We identified subgroups of patients with obesity, HTN and dyslipidemia. Informed consents were obtained from all participants. The study design, within the biological collection, did not include patient matching.

Blood was collected on citrated tubes according to MISEV-2018 guidelines [33]. Platelet-free plasma was prepared by double centrifugation within 4h and stored at -80°C.

**Inclusion parameters:**

- Age>18 years

- T2D group: Type 2 diabetes

- Control group: HTN (Systolic blood pressure ≥140mmHg and/or Diastolic blood pressure ≥90mmHg or anti-hypertensive treatment) or dyslipidemia AND no diabetes.

**Non-inclusion parameters:**

- SCD or intermedia/major β-thalassemia

- Recent anticoagulant treatment (curative dose)

- Pregnancy or postpartum (40 days after delivery)

- Recent alcohol (<10 hours)/coffee (<3 hours)/tobacco (<36 hours) consumption

- Active hepatitis B, C or HIV infection

- Active cancer or hematologic malignancy

- Coagulation disorders

- Active inflammatory or infectious disease

- Recent (<3 months) venous or arterial thrombotic event

- Adult persons under protection measures

- Non affiliation to a social security scheme

- Enrollment in another research protocol.

**Blood sample collection**

Venous blood was collected in trisodium-citrate-coated tubes (BD Biosciences, Le Pont-de-Claix, France). Plasma was prepared according to MISEV-2018 guidelines (33), by two consecutive centrifugation steps at 2500-g, at room temperature for 15 min, in order to obtain platelet-free plasma within 4h of blood collection: one step to remove blood cells and a second step to remove remaining platelets. Plasma was aliquoted, stored at -80°C and aliquots were thawed once for analysis.

**Flow cytometry for extracellular vesicles**

Quantification of EV was performed by fluorescence-assisted cell sorting (FACS) using a protocol adapted for EV, as previously reported (Camus, 2012; Camus, 2015; Merle, 2018; Hariri, 2019). Plasma or cell supernatants were thawed at room temperature for 5 minutes before being placed on ice. To avoid swarm detection, plasma was diluted 20-fold with PBS (1.45 mM phosphate and 154 mM sodium chloride; pH 7.4, 0.1 µm filtered), to less than 5000 events/s.

To establish cellular origin and phosphatidylserine (PS) exposure of EV, we prepared a labeling mix: 20 µL annexin-A5-FITC +10 µL anti-CD235a or isotype, in 1 mL PBS +20 µL (0.5 M Ca2+). Before mixing, antibodies were filtered on a Ceveron® Microparticle Filtration Unit (MFU) 500, at room temperature to remove potential antibody aggregates. Samples were labeled by addition of 10 µL of sample with 90 µL of PBS, and 100 µL of labeling mix (final = 5 mM Ca2+). Samples were then incubated in the dark for 40 minutes at room temperature.

Flow-Count™ Fluorospheres (Beckman-Coulter, Villepinte, France; 10 µm diameter) were then added immediately before EV detection using a LSR-Fortessa™ Cell Analyzer (BD Biosciences). Samples (220 μl of mix, total) were analyzed for up to 1 minute or up to 2000 Fluorospheres, with a flow rate set at 12 µL/minute, using side light scatter (SSc) for triggering.

Data were analyzed with the FlowJo software (Version 10; FlowJo LLC, via BD Biosciences). All sample events were visualized in a first flow cytometry analysis window displaying Forward scatter (FSc) by Side light scatter (SSc). For EV-size events, a gate was set on Megamix-Plus-SSC^TM^ calibration microbeads (Biocytex, Marseille, France) in a dedicated suspension, covering diameters of 0.1 µm – 0.3 µm – 0.5 µm and 0.9 µm, and excluding the 10 μm size range where Flow-count Fluorospheres are detected. Quantification Flow-Count™ Fluorospheres were observed in the upper right quadrant of the FSc by SSc window and used as reference for the sample volume analyzed.

Size-gated events were then visualized in a second analysis window displaying FITC by APC fluorescence. To estimate CD235a- and PS-positive EV/μL plasma, fluorescence thresholds were set to exclude signals generated by a sample pool labeled with control IgG or annexin-A5 in the absence of Ca2+ (negative controls), and to include fluorescent events contained by a pool of samples labeled with anti-CD235a antibody and annexin-A5 in the presence of Ca^2+^ (positive controls).

The number of labeled, EV-size events (nEV) found in the relevant quadrants were used in the following formula, based on the concentration of each particular stock of counting Fluorospheres:

EV concentration (EV/μL) = (nEV / volume) x (nFluoS-total / nFluoS-detected).

nEV = Number of events from the sample that appear within the EV size gate (FSc xSSc) and within the relevant label gate.

volume = Volume (μl) of sample added to the mix.

mix = Sample +buffer +rhAnnexin-A5 +antibodies +counting FluoS.

nFluoS-detected = Number of Fluorospheres detected in sample volume read (factual).

nFluoS-total = Number of Fluorospheres (total) inserted into the mix; ie. volume (μl) of Fluorospheres inserted into the mix x Fluorosphere stock concentration (FluoS/μl).

For PS+ annexin-V-labeled EV (single labeled), we added up events appearing in the upper left and upper right quadrants of the fluorescence window. For CD235a-labeled EV (single labeled), we added up events appearing in the upper right and lower right quadrants. For PS+ and annexin-V-EV (double labeled), we used the number of events appearing in upper right quadrant only.
