# Supplementary material for: Low grade intravascular hemolysis associates with peripheral nerve injury in type 2 diabetes
Source: PLoS One. 2022 Oct 17;17(10):e0275337. doi: 10.1371/journal.pone.0275337 (PMC9576093; doi:10.1371/journal.pone.0275337)
Supplement: S2 File — (PDF) [file pone.0275337.s002.pdf]

Comité de Protection des Personnes
Ile-de-France VII

Hôpital de Bicêtre – 78 rue du général Leclerc – 94275 Le Kremlin Bicêtre Cedex
Président : Jacques CARRE – Secrétaire : Brigitte PILATE

Projet de Recherche N° : ***C0-12-003*** Kremlin Bicêtre le 30 mai 2012

Le Comité a été saisi le 9 janvier 2012
par Monsieur JEANJEAN pour le compte l'INSERM – DR Paris 5 – CS 51419 – 2 rue d'Alésia – 75014 PARIS
d'un dossier intitulé :
« DOSSIER DE DECLARATION DES ACTIVITES DE CONSERVATION ET DE PREPARATION D'ELEMENTS ISSUS DU CORPS HUMAIN EXERCEES POUR LES BESOINS DES PROGRAMMES DE RECHERCHE DE L'INSERM : recherche Hème et Microparticules chez le patient diabétique organisée par l'Inserm et l'Assistance Publique-Hôpitaux de Paris »
(Référence du promoteur : N° : DC-2011-1480 – responsable scientifique : Docteur BLANC-BRUDE – INSERM UMRs-970, ParCC, Hôpital Européen Georges Pompidou – 56 rue Leblanc – 75015 PARIS)

Le comité a examiné les informations relatives à ce dossier lors de la séance du 1er février 2012 avec lettre d'information pour le patient majeur version du 20/12/2011

Membres présents lors de la délibération de votre protocole

Premier Collège :
- Recherche biomédicale : Monsieur V. GAJDOS, pédiatre (T), Madame A. LAPLANCHE, épidémiologiste (T), Monsieur M. PUCHEAULT (T) et Monsieur M. BOTTLAENDER (S)
- Médecin généraliste : Monsieur A. DUBOIS (S)
- Pharmacien : Madame A. M. TABURET (T)
- Infirmière : Madame C. ASTOUL (T)

Deuxième collège :
- Ethique : Monsieur J. CARRE (T)
- Psychologue : Madame S. SCHWAB (T)
- Travailleur social : Madame A. M PETIT (T)
- Juristes : Madame V. A. LAFOY (T) et Madame F. BOISSY (T)
- Associations agréées : Madame A. LABBE (T) et Monsieur COTTET (S)

Le comité après avoir délibéré en conformité avec les textes règlementaires a adopté les conclusions suivantes :
- Les renseignements prescrits par la loi sont renseignés de manière satisfaisante (art R 1243 – 51 CSP et art R 1243 – 53 CSP) y compris concernant les formalités relatives à la mise en œuvre du traitement des données,
- La procédure d'information des patients est décrite,
- L'organisme assure pour les besoins de ses propres programmes de recherche la préparation, la conservation, l'utilisation d'éléments du corps humain et la constitution de collections d'échantillons biologiques,
- La cession à un autre organisme de recherche n'est pas envisagée,
- La destruction des échantillons est prévue à la fin de la recherche

Jacques CARRE
Président du C.P.P. I.D.F. VII
