# Supplementary material for: Low grade intravascular hemolysis associates with peripheral nerve injury in type 2 diabetes
Source: PLoS One. 2022 Oct 17;17(10):e0275337. doi: 10.1371/journal.pone.0275337 (PMC9576093; doi:10.1371/journal.pone.0275337)
Supplement: S4 File — (PDF) [file pone.0275337.s004.pdf]

Commitee for patient protection – Ile-de-France

Hôpital de Bicêtre – 78 rue du Général Leclerc – 94275 Le Kremlin-Bicêtre Cedex
President: Jacques CARRE – Secretary: Brigitte PILATE

Research Project n°: ***C0-12-003*** Kremlin-Bicêtre May 30, 2012

The committee deliberated on January 9, 2012, sollicited by Mister JEANJEAN, for the benefit of INSERM – DR Paris 5 – CS 51419 – N°2 rue d'Alesia – 75014 PARIS, on a proposal called:

« **Form to declare activities of conservation and preparation of elements derived from the Human body, for the needs of research programs of INSERM: research project Heme and microparticles in diabetic patients, organized by INSERM and Assistance Publique–Hôpitaux de Paris.** »
(Reference of the promoter: n°: DC-2011-1480 – Scientific officer: Dr BLANC-BRUDE INSERM UMRs-970, ParCC, Hôpital Européen Georges Pompidou – 56 rue Leblanc – 75015 PARIS.)

The committee examined the information pertaining to this request during the session of February 1st, 2012, with an information letter for adult patients, version of 20 / 12 / 2011.

Members present during the deliberation on your protocol,
First college:

- Biomedical research: Mister V. GAJDOS, pediatrician (T), Mme A LAPLANCHE, epidemiologist (T), Mister M. PUCHEULT (T) and Mister M. BOLLAENDER (S)
- General Practitioner: Mister A DUBOIS (S)
- Pharmacian: Madam A. M TABURET (T)
- Nurse: Madam C. ASTOUL (T)

Second college:

- Ethics: Mister J. CARRE (T)
- Psychologist: Madam A. M. PETIT (T)
- Lawyers: Madam V.A. LAFOY (T) and Madam F. BOISSY (T)
- Partner associations: Madam A. LABBE (T) and Mister COTTET (S)

The committee agreed on the following position, after deliberating based on reglementary reference texts:

- Information elements imposed by law were provided in a satisfactory manner (art R1243 – 51 CSP and art R1243 – 53 CSP), including formal information related to the implementation of data treatment,
- The procedure to inform patients is described,
- The institution will cover the needs of its own research programs for the preparation, conservation and use of elements derived from the Human body, as well as the constitution of biological collections,
- The possible donation to another research institution was not envisaged,
- The destruction of the samples is planned when research is completed.

Jacques CARRE,
President of the C.P.P. I.D.F. VII
