# Supplementary material for: Low grade intravascular hemolysis associates with peripheral nerve injury in type 2 diabetes
Source: PLoS One. 2022 Oct 17;17(10):e0275337. doi: 10.1371/journal.pone.0275337 (PMC9576093; doi:10.1371/journal.pone.0275337)
Supplement: S1 Table — The table lists the clinical data that were significantly associated with T2D in cohort Diabelyse, in univariate and multivariate analyses. Those included classical traits of T2D like high Hb1Ac, high fasting glycemia, lower bilirubin and higher leukocyte levels, but also our plasma marker of IVH, Abs398 (related to heme). In mutivariate analyses$, Abs575 was not significant, suggesting that Abs398 may be a more robust biomarker. The average RBC volume was also reduced in T2D. Data are median (25th–75th percentiles) or n (%). *P<0.05. $ After adjustment for age, sex, obesity, dyslipidemia and HTN status. Data for bilirubin and LDH were only collected for 7% patients. BMI, body mass index; Obesity, BMI ≥30kg/m2; eGFR, estimated glomerular filtration rate; AST, aspartate aminotransferase; ALT, alanine aminotransferase; RBC, red blood cells; MCV, mean corpuscular volume; MCHC, mean corpuscular hemoglobin concentration; LDH, lactate dehydrogenase; CRP, C reactive protein. (DOCX) [file pone.0275337.s005.docx]

**SUPPORTING INFORMATION**

**Low Grade Intravascular Hemolysis Associates with Peripheral Nerve Injury**

**in Type 2 Diabetes**

Sylvain Le Jeune, MD ^1,2^ ; Sihem Sadoudi, PhD ^1^ ; Dominique Charue, MSc ^1^ ; Salwa Abid, MSC ^1^ ; Jean-Michel Guigner, PhD ^3^ ; Dominique Helley, MD PhD ^1,4^ ; Hélène Bihan, MD PhD ^5^ ; Camille Baudry, MD ^6^ ; Hélène Lelong, MD PhD ^7^ ; Tristan Mirault, MD PhD ^1,8^ ; Eric Vicaut, MD PhD ^1,9^ ; Robin Dhote, MD PhD ^2^ ; Jean-Jacques Mourad, MD PhD ^10^ ; Chantal M. Boulanger, PhD ^1^; Olivier P. Blanc-Brude, PhD ^1^.

**Short title:**

Intravascular Hemolysis is a component of type 2 diabetes associated with peripheral neuropathy.

**Key Words:**

Type 2 diabetes, Intravascular hemolysis, Red blood cells, Hemoglobin, Heme, Extracellular vesicles, Peripheral Neuropathy.

**Manuscript data:** Le Jeune/2022/Version 1

**Correspondence:**

Olivier Blanc-Brude

Paris Center for Cardiovascular Research - Inserm U970

Hôpital Européen Georges Pompidou,

56 rue Leblanc, F-75015 PARIS, France

Tel : +33 / 1 53 98 80 61

e-mail : [olivier.blanc-brude@inserm.fr](mailto:olivier.blanc-brude@inserm.fr)

**Table S1. Baseline clinical and biological characteristics of patients included in the DIABELYSE study**

|  | **Controls  n=65** | **T2D  n=109** | ***p (univariate)*** | ***p (multivariate)^$^*** |
| --- | --- | --- | --- | --- |
| Abs398 | 0.324 (0.243-0.393) | 0.508 (0.341-0.547) | <0.001* | **0.020*** |
| Abs575 | 0.025 (0.019-0.042) | 0.040 (0.024-0.056) | 0.003* | 0.241 |
| Age (years) | 48 (42-58) | 62 (54-69) | <0.001* | - |
| Male sex | 34 (56.7) | 44 (40.4) | 0.039* | - |
| BMI (kg/m^2^) | 28 (25.9-31.3) | 31.2 (27.7-35.1) | <0.001* | - |
| Obesity | 21 (35.6) | 66 (61.7) | <0.001* | - |
| Dyslipidemia | 20 (33.9) | 87 (80.6) | <0.001* | - |
| Hypertension | 37 (56.1) | 79 (86.5) | 0.004* | - |
| HbA1C (%) | 5.5 (5.2-5.9) | 8 (7.2-9.6) | <0.001* | **<0.001*** |
| Fasting glycemia (g/l) | 0.94 (0.87-0.98) | 1.39 (1.07-1.84) | <0.001* | **<0.001*** |
| eGFR (CKD) (ml/min) | 94 (80-109) | 76 (54.5-99) | <0.001* | 0.196 |
| RBC (10^6^/mm^3^) | 4.82 (4.48-5.12) | 4.73 (4.32-5.11) | 0.14 | 0.853 |
| Hemoglobin (g/dl) | 13.6 (12.85-14.6) | 13 (12.1-14.1) | 0.011* | 0.716 |
| Hematocrit (%) | 41.9 (40.2-44.27) | 39.4 (36.5-41.7) | <0.001* | 0.195 |
| MCV (u^3^) | 85.3 (83.6-89.4) | 82.9 (80.3-88.25) | 0.049* | **0.027*** |
| MCHC (%) | 33.4 (31.9-34.1) | 33.5 (32.75-34.15) | 0.266 | 0.176 |
| Leukocytes (10^3^/mm^3^) | 5.47 (4.56-6.2) | 7 (6-8.1) | <0.001* | **0.005*** |
| CRP (mg/l) | 0.48 (0-3) | 3 (1-7) | <0.001* | 0.062 |
| AST (U/l) | 23 (18-30) | 22 (18-26) | 0.485 | 0.719 |
| ALT (U/l) | 25.5 (19.2-30.7) | 24 (18-31) | 0.866 | 0.24 |
| Serum iron (umol/l) | 13 (13-13.5) | 12 (10-15) | 0.560 | 0.35 |
| Bilirubin (umol/l) | 8.5 (8-12.25) | 5.5 (4-7.25) | 0.004* | **0.036*** |
| LDH (U/l) | 392 (357-437.5) | 416 (380.5-465.5) | 0.471 | NA |

Data are median (25^th^-75^th^ percentiles) or n (%). **P*<0.05. ^$^ After adjustment for age, sex, obesity, dyslipidemia and HTN status. Data for bilirubin and LDH were only collected for 7% patients. BMI, body mass index; Obesity, BMI ≥30kg/m^2^; eGFR, estimated glomerular filtration rate; AST, aspartate aminotransferase; ALT, alanine aminotransferase; RBC, red blood cells; MCV, mean corpuscular volume; MCHC, mean corpuscular hemoglobin concentration; LDH, lactate dehydrogenase; CRP, C reactive protein.
