# Supplementary material for: Low grade intravascular hemolysis associates with peripheral nerve injury in type 2 diabetes
Source: PLoS One. 2022 Oct 17;17(10):e0275337. doi: 10.1371/journal.pone.0275337 (PMC9576093; doi:10.1371/journal.pone.0275337)
Supplement: S2 Table — The table reveals that only two independent variables were significantly associated to Diabetic Peripheral Sensory Neuropathy in cohort Diabelyse, namely Abs398 and Abs575. In contrast, other parameters related to RBC did not, including RBC counts, serum iron, plasma bilirubin, or classical risk factors. Data are median (25th–75th percentiles) or n (%). *p<0.05. $ after adjustment with factors classically involved in the pathogenesis of neuropathy in T2D: HbA1C, T2D duration, age and sex. Data for bilirubin and LDH were only collected for 7% patients. Obesity, BMI≥30kg/m2; eGFR, estimated glomerular filtration rate; RBC, red blood cells; MCV, mean corpuscular volume; MCHC, mean corpuscular hemoglobin concentration; LDH, lactate dehydrogenase; CRP, C reactive protein. (DOCX) [file pone.0275337.s006.docx]

**SUPPORTING INFORMATION**

**Low Grade Intravascular Hemolysis Associates with Peripheral Nerve Injury**

**in Type 2 Diabetes**

Sylvain Le Jeune, MD ^1,2^ ; Sihem Sadoudi, PhD ^1^ ; Dominique Charue, MSc ^1^ ; Salwa Abid, MSC ^1^ ; Jean-Michel Guigner, PhD ^3^ ; Dominique Helley, MD PhD ^1,4^ ; Hélène Bihan, MD PhD ^5^ ; Camille Baudry, MD ^6^ ; Hélène Lelong, MD PhD ^7^ ; Tristan Mirault, MD PhD ^1,8^ ; Eric Vicaut, MD PhD ^1,9^ ; Robin Dhote, MD PhD ^2^ ; Jean-Jacques Mourad, MD PhD ^10^ ; Chantal M. Boulanger, PhD ^1^; Olivier P. Blanc-Brude, PhD ^1^.

**Short title:**

Intravascular Hemolysis is a component of type 2 diabetes associated with peripheral neuropathy.

**Key Words:**

Type 2 diabetes, Intravascular hemolysis, Red blood cells, Hemoglobin, Heme, Extracellular vesicles, Peripheral Neuropathy.

**Manuscript data:** Le Jeune/2022/Version 1

**Correspondence:**

Olivier Blanc-Brude

Paris Center for Cardiovascular Research - Inserm U970

Hôpital Européen Georges Pompidou,

56 rue Leblanc, F-75015 PARIS, France

Tel : +33 / 1 53 98 80 61

e-mail : [olivier.blanc-brude@inserm.fr](mailto:olivier.blanc-brude@inserm.fr)

**Table S2. Variables associated to Diabetic Peripheral Sensory Neuropathy**

|  | **No neuropathy** | **Neuropathy** | ***p (univariate)*** | ***p (multivariate)^$^*** |
| --- | --- | --- | --- | --- |
| Insulin | 31 (47.7) | 40 (67.8) | **0.029*** | 0.103 |
| **Abs398** | 0.478  (0.334-0.699) | 0.617  (0.404-0.808) | **0.046*** | **0.049*** |
| **Abs575** | 0.038  (0.023-0.050) | 0.049  (0.031-0.057) | **0.047*** | **0.045*** |
| Obesity | 35 (53.8) | 38 (65.5) | 0.203 | 0.945 |
| Dyslipidemia | 52 (80) | 49 (83.1) | 0.818 | 0.893 |
| Hypertension | 47 (72.3) | 48 (81.4) | 0.290 | 0.898 |
| Smoking | 7 (10.8) | 7 (11.9) | 1 | 0.509 |
| Nephropathy | 31 (49.2) | 37 (67.3) | 0.062 | 0.133 |
| eGFR (CKD EPI) (ml/min) | 86 (60-100) | 66 (46-91) | **0.004*** | **0.042*** |
| Microalbuminuria | 17 (28.3) | 17 (35.4) | 0.532 | 0.540 |
| Retinopathy | 28 (45.2) | 36 (63.2) | 0.066 | 0.067 |
| Laser for retinopathy | 4 (6.5) | 19 (33.3) | **<0.001*** | **<0.001*** |
| Macroangiopathy | 7 (10.8) | 20 (33.9) | **0.002*** | **0.047*** |
| Hemoglobin (g/dl) | 13 (12.3-14.3) | 12.6 (11.5-13.7) | 0.078 | 0.804 |
| Hematocrit (%) | 39.3 (36.8-42.4) | 37.9 (34.5-41) | 0.104 | 0.494 |
| RBC (10^6^/mm^3^) | 4.6 (4.3-5.1) | 4.6 (4.1-5) | 0.463 | 0.853 |
| MCV (u^3^) | 83.8 (80.9-88.5) | 83 (80.9-89) | 0.960 | 0.611 |
| MCHC (%) | 33.5 (32.6-34.1) | 33.3 (32.7-34) | 0.432 | 0.568 |
| Leukocytes (10^3^/mm^3^) | 6.9 (5.9-8.1) | 7.2 (6.1-8.6) | 0.215 | 0.476 |
| CRP (mg/l) | 2.5 (1-8) | 4 (1.3-8) | 0.165 | 0.732 |
| Serum Iron (umol/l) | 12 (10-15) | 12 (9-14.5) | 0.427 | 0.879 |
| Bilirubin (umol/l) | 8.5 (5.5-10.5) | 9 (7.5-12.5) | 0.382 | 0.361 |
| LDH (U/l) | 447.5  (352.5-498) | 218  (184.5-290) | 0.214 | 0.885 |

Data are median (25^th^-75^th^ percentiles) or n (%). **p*<0.05. ^$^ after adjustment with factors classically involved in the pathogenesis of neuropathy in T2D: HbA1C, T2D duration, age and sex. Data for bilirubin and LDH were only collected for 7% patients. Obesity, BMI≥30kg/m^2^; eGFR, estimated glomerular filtration rate; RBC, red blood cells; MCV, mean corpuscular volume; MCHC, mean corpuscular hemoglobin concentration; LDH, lactate dehydrogenase; CRP, C reactive protein.
