# Supplementary material for: Low grade intravascular hemolysis associates with peripheral nerve injury in type 2 diabetes
Source: PLoS One. 2022 Oct 17;17(10):e0275337. doi: 10.1371/journal.pone.0275337 (PMC9576093; doi:10.1371/journal.pone.0275337)
Supplement: S3 Table — The table reveals that the levels of PS+ CD235+ EV were not significantly associated to any of the markers of vascular injury in T2D cohort Diabelyse (all OR >0.98; all p>0.065). Data are median (25th–75th percentiles), or Odds Ratios and their 95% confidence intervals after adjustment for age and sex. Obesity, BMI≥30kg/m2; eGFR, estimated glomerular filtration rate. PS, phosphatidylserine; EV, extracellular vesicles; CD235 is also glycophorin-235. (DOCX) [file pone.0275337.s007.docx]

**SUPPORTING INFORMATION**

**Low Grade Intravascular Hemolysis Associates with Peripheral Nerve Injury**

**in Type 2 Diabetes**

Sylvain Le Jeune, MD ^1,2^ ; Sihem Sadoudi, PhD ^1^ ; Dominique Charue, MSc ^1^ ; Salwa Abid, MSC ^1^ ; Jean-Michel Guigner, PhD ^3^ ; Dominique Helley, MD PhD ^1,4^ ; Hélène Bihan, MD PhD ^5^ ; Camille Baudry, MD ^6^ ; Hélène Lelong, MD PhD ^7^ ; Tristan Mirault, MD PhD ^1,8^ ; Eric Vicaut, MD PhD ^1,9^ ; Robin Dhote, MD PhD ^2^ ; Jean-Jacques Mourad, MD PhD ^10^ ; Chantal M. Boulanger, PhD ^1^; Olivier P. Blanc-Brude, PhD ^1^.

**Short title:**

Intravascular Hemolysis is a component of type 2 diabetes associated with peripheral neuropathy.

**Key Words:**

Type 2 diabetes, Intravascular hemolysis, Red blood cells, Hemoglobin, Heme, Extracellular vesicles, Peripheral Neuropathy.

**Manuscript data:** Le Jeune/2022/Version 1

**Correspondence:**

Olivier Blanc-Brude

Paris Center for Cardiovascular Research - Inserm U970

Hôpital Européen Georges Pompidou,

56 rue Leblanc, F-75015 PARIS, France

Tel : +33 / 1 53 98 80 61

e-mail : [olivier.blanc-brude@inserm.fr](mailto:olivier.blanc-brude@inserm.fr)

**Table S3. Associations of PS+ and CD235a+ large EV with cardiovascular risk factors, or micro- and macrovascular complications in T2D patients**

|  | **PS+ CD235a+ large EV** | | | |
| --- | --- | --- | --- | --- |
|  | **No** | **Yes** | ***OR*** | ***p*** |
| Hypertension | 1427 (957;2138) | 1554 (914;2221) | 1.00 (0.99;1.00) | 0.713 |
| Dyslipidemia | 1459 (1043;2353) | 1510 (906;2141) | 1.00 (0.99;1.00) | 0,760 |
| Obesity | 1428 (1013;2864) | 1598 (1172;2141) | 0.99 (0.99;1.00) | 0,103 |
| Microangiopathy | 2138 (1469;3286) | 1440 (852;2141) | 0.99 (0.99;1.00) | 0.099 |
| Neuropathy | 1469 (1276;2150) | 1554 (695;2193) | 1.00 (0.99;1.00) | 0.693 |
| Nephropathy | 1510 (1043;2141) | 1369 (754;2188) | 0.99 (0.99;1.00) | 0.550 |
| Microalbuminuria | 1486 (921;2140) | 1854 (960;2517) | 1.00 (0.99;1.00) | 0.778 |
| Proteinuria | 1766 (1146;2141) | 1354 (680;2353) | 0.99 (0.99;1.00) | 0.666 |
| eGFR <60mL/min | 1498 (951;2169) | 1354 (680;2023) | 0.99 (0.99;1.00) | 0.324 |
| Retinopathy | 1411 (1008;2168) | 1810 (951;2300) | 0.99 (0.99;1.00) | 0.292 |
| Maculopathy | 1510 (921;2178) | 2356 (2248;2464) | 1.00 (0.99;1.00) | 0.809 |
| Laser for retinopathy | 1369 (860;2159) | 2140 (1598;2353) | 0.99 (0.99;1.00) | 0.725 |
| Cardiovascular disease | 1598 (1354;2177) | 680 (618;786) | 0.98 (0.01;851) | 0.067 |
| Coronary heart disease | 1598 (1354;2177) | 680 (618;786) | 0.98 (0.01;851) | 0.067 |
| Stroke | 1510 (982;2159) | 555 (555;555) | NA | 0.537 |
| Peripheral arterial disease | 1510 (982;2159) | 555 (555;555) | NA | 0.537 |

Data are median (25^th^-75^th^ percentiles), or Odds Ratios and their 95% confidence intervals after adjustment for age and sex. Obesity, BMI≥30kg/m^2^; eGFR, estimated glomerular filtration rate. PS, phosphatidylserine; EV, extracellular vesicles; CD235a is also named glycophorin-235a.
