# Supplementary material for: Low grade intravascular hemolysis associates with peripheral nerve injury in type 2 diabetes
Source: PLoS One. 2022 Oct 17;17(10):e0275337. doi: 10.1371/journal.pone.0275337 (PMC9576093; doi:10.1371/journal.pone.0275337)
Supplement: S1 Checklist — (DOCX) [file pone.0275337.s008.docx]

STROBE Statement—checklist of items that should be included in reports of observational studies

|  | Item No. | Recommendation | Page  No. | Relevant text from manuscript |
| --- | --- | --- | --- | --- |
| **Title and abstract** | 1 | (*a*) Indicate the study’s design with a commonly used term in the title or the abstract | 2 | We characterized RBC-degradation products and vesiculation in a case-control study of 109 T2D patients and 65 control subjects. |
|  |  | (*b*) Provide in the abstract an informative and balanced summary of what was done and what was found | 2 | See manuscript Abstract |
| Introduction | | | |  |
| Background/rationale | 2 | Explain the scientific background and rationale for the investigation being reported | 3-4 | See manuscript Introduction section |
| Objectives | 3 | State specific objectives, including any prespecified hypotheses | 4 | See end of manuscript Introduction section |
| Methods | | | |  |
| Study design | 4 | Present key elements of study design early in the paper | 4 | See manuscript Materials and methods section, first two paragraphs |
| Setting | 5 | Describe the setting, locations, and relevant dates, including periods of recruitment, exposure, follow-up, and data collection | 4 | See manuscript Materials and methods section, first two paragraphs (and supplementary information). |
| Participants | 6 | (*a*) *Cohort study*—Give the eligibility criteria, and the sources and methods of selection of participants. Describe methods of follow-up  *Case-control study*—Give the eligibility criteria, and the sources and methods of case ascertainment and control selection. Give the rationale for the choice of cases and controls  *Cross-sectional study*—Give the eligibility criteria, and the sources and methods of selection of participants | 4 | See manuscript Materials and methods section, first paragraph (and supplementary information).  We compared diabetic and non-diabetic patients as controls. |
|  |  | (*b*) *Cohort study*—For matched studies, give matching criteria and number of exposed and unexposed  *Case-control study*—For matched studies, give matching criteria and the number of controls per case |  |  |
| Variables | 7 | Clearly define all outcomes, exposures, predictors, potential confounders, and effect modifiers. Give diagnostic criteria, if applicable | 5 – 6 - 7 | See manuscript Materials and methods section, biomarkers and vascular injury paragraphs. |
| Data sources/ measurement | 8* | For each variable of interest, give sources of data and details of methods of assessment (measurement). Describe comparability of assessment methods if there is more than one group | 5 – 6 - 7 | See manuscript Materials and methods section, biomarkers and vascular injury paragraphs. |
| Bias | 9 | Describe any efforts to address potential sources of bias | Tables 1 & 2 p14 &15, p16 | Potential sources of bias were addressed by reporting adjusted multivariate analyses whenever possible (Results and tables), and by comments (Discussion). |
| Study size | 10 | Explain how the study size was arrived at | Suppl. | Study size number was not calculated as no data was available to formulate a hypothesis: No measurements of plasma absorbance and no measurements of intravascular hemolysis in T2 diabetic patients. Such biomarkers investigated in genetic blood disorders are difficult to transpose to T2 diabetes, where subclinial hemolysis was expected. |

Continued on next page

| Quantitative variables | 11 | Explain how quantitative variables were handled in the analyses. If applicable, describe which groupings were chosen and why | 7 – 8 | See Methods section, Statistics paragraph |
| --- | --- | --- | --- | --- |
| Statistical methods | 12 | (*a*) Describe all statistical methods, including those used to control for confounding | 7 – 8 | See Methods section, Statistics paragraph. Multivariate logistic regression analysis was performed with adjustment for age, sex, obesity, dyslipidemia and HTN status |
|  |  | (*b*) Describe any methods used to examine subgroups and interactions | 7 – 8 | See Methods section, Statistics paragraph. Biomarkers levels in specific T2D patient subgroups were compared by Wilcoxon signed-rank test, and potential association of log-transformed plasma absorbance with cardiovascular risk factors, vascular complications and treatments in T2D patients were assessed using multivariate logistic regressions adjusted for age and sex. |
|  |  | (*c*) Explain how missing data were addressed | 8 | Missing data were considered as negligible (<9%), except for MCV (31%), MCHC (31%), fasting glycemia (66%). Only a few datapoints could be collected for bilirubin (7%) and LDH (6%) levels. Missing data were addressed by removing the data via the dedicated R software function. |
|  |  | (*d*) *Cohort study*—If applicable, explain how loss to follow-up was addressed  *Case-control study*—If applicable, explain how matching of cases and controls was addressed  *Cross-sectional study*—If applicable, describe analytical methods taking account of sampling strategy | 5 | The study design, within the biological collection, did not include patient matching. Statistical analyses included multivariate analyses, with adjustment for age and sex, as well as cardiovascular risk factors for T2D versus non-T2D patients |
|  |  | (*e*) Describe any sensitivity analyses | 5 | Sensitivity analyses were not performed in this study (missing data not imputed). |
| Results | | | | |
| Participants | 13* | (a) Report numbers of individuals at each stage of study—eg numbers potentially eligible, examined for eligibility, confirmed eligible, included in the study, completing follow-up, and analysed | 4 | NA - We performed a case-control study nested in the biological collection, basing inclusion on the existence of biological samples and patient records for the relevant clinical and biological data for cases and controls. |
|  |  | (b) Give reasons for non-participation at each stage |  | NA – see above |
|  |  | (c) Consider use of a flow diagram |  | NA – see above |
| Descriptive data | 14* | (a) Give characteristics of study participants (eg demographic, clinical, social) and information on exposures and potential confounders | Supplements | Supplementary table 1, for cases and controls. |
|  |  | (b) Indicate number of participants with missing data for each variable of interest | 14 - 15 | The number of data (and missing data) was made readily available with a dedicated column for all parameters in Tables 1, 2 and 3.  Nb: Note inserted below tables: Data for bilirubin and LDH were only collected for 7% patients. |
|  |  | (c) *Cohort study*—Summarise follow-up time (eg, average and total amount) |  | NA |
| Outcome data | 15* | *Cohort study*—Report numbers of outcome events or summary measures over time |  | NA |
|  |  | *Case-control study—*Report numbers in each exposure category, or summary measures of exposure | 15 - 16 | Numbers reported in all tables, for cases and controls. |
|  |  | *Cross-sectional study—*Report numbers of outcome events or summary measures |  |  |
| Main results | 16 | (*a*) Give unadjusted estimates and, if applicable, confounder-adjusted estimates and their precision (eg, 95% confidence interval). Make clear which confounders were adjusted for and why they were included | 15 - 16 | Numbers reported in tables, for cases and controls. Confounders listed below all relevant tables. |
|  |  | (*b*) Report category boundaries when continuous variables were categorized | 7, 15 - 16 | Renal failure (eGFR levels), dyslipidemia, microalbuminuria and proteinuria thresholds were described in relevant paragraphs. |
|  |  | (*c*) If relevant, consider translating estimates of relative risk into absolute risk for a meaningful time period |  | NA |

Continued on next page

| Other analyses | 17 | Report other analyses done—eg analyses of subgroups and interactions, and sensitivity analyses | 11 – Suppl. | Next, we assessed the association of our IVH biomarkers with clinical data and vascular injury in T2D patients, identifying groups with obesity, HTN and dyslipidemia. Clinical laboratory data revealed a 3.4% drop in RBC volume (MCV) in T2D, overall (85.3 vs. 82.9 μm^3^, *p*=0.027), using multivariate analysis adjusted for age, sex, obesity, HTN and dyslipidemia **(Supplementary table 1)**. |
| --- | --- | --- | --- | --- |
| Discussion | | | | |
| Key results | 18 | Summarise key results with reference to study objectives | 16 | See discussion section |
| Limitations | 19 | Discuss limitations of the study, taking into account sources of potential bias or imprecision. Discuss both direction and magnitude of any potential bias | 17 | Larger, multicentric prospective studies with higher statistical power are now needed to clarify the association of heme-related absorbance with microvascular complications in T2D, and their relevance to stratify T2D patients at risk of developing peripheral neuropathy. |
| Interpretation | 20 | Give a cautious overall interpretation of results considering objectives, limitations, multiplicity of analyses, results from similar studies, and other relevant evidence | 16 – 17 – 18 | See Discussion section |
| Generalisability | 21 | Discuss the generalisability (external validity) of the study results | 17 | Larger, multicentric prospective studies with higher statistical power are now needed to clarify the association of heme-related absorbance with microvascular complications in T2D, and their relevance to stratify T2D patients at risk of developing peripheral neuropathy. |
| Other information | |  | | |
| Funding | 22 | Give the source of funding and the role of the funders for the present study and, if applicable, for the original study on which the present article is based | 19 | Project ‘RépaDia’ of the Fondation de France to OBB, Project ‘BiFace’ of the Fondation pour la Recherche Médicale to OBB, Project ‘Cardannex’ of Inserm Transfert to OBB. The funders had no bearing on the conduct of the study. |

*Give information separately for cases and controls in case-control studies and, if applicable, for exposed and unexposed groups in cohort and cross-sectional studies.

**Note:** An Explanation and Elaboration article discusses each checklist item and gives methodological background and published examples of transparent reporting. The STROBE checklist is best used in conjunction with this article (freely available on the Web sites of PLoS Medicine at http://www.plosmedicine.org/, Annals of Internal Medicine at http://www.annals.org/, and Epidemiology at http://www.epidem.com/). Information on the STROBE Initiative is available at www.strobe-statement.org.
