# Supplementary material for: Low grade intravascular hemolysis associates with peripheral nerve injury in type 2 diabetes
Source: PLoS One. 2022 Oct 17;17(10):e0275337. doi: 10.1371/journal.pone.0275337 (PMC9576093; doi:10.1371/journal.pone.0275337)
Supplement: Attachment — Submitted filename: Hemolysis in T2D (LeJeune-Blanc-Brude)- Response Reviewers.docx [file pone.0275337.s010.docx]

Dear editors of **PLOS One**,	September **09**, 2022

Please find herewith the revised version of our new manuscript, which we wish to submit to your consideration for publication in PLOS One as a regular article entitled:

**Low Grade Intravascular Hemolysis Associates with Peripheral Nerve Injury in Type 2 Diabetes.**

**Response to reviewers on page 2-3, in blue**

Intravascular hemolysis is a pathological event that occurs in hematological diseases, like sickle cell disease. It involves the release of toxic products into the circulation by injured red blood cells, such as hemoglobin, heme and heme-loaded extracellular vesicles.

We demonstrated previously that red blood cell-derived extracellular vesicles, loaded with heme, are typical of intravascular hemolysis. They damage cultured endothelial cells *in vitro*, compromise vascular function, and lead to acute vaso-occlusive events [Camus, Blood 2012; Camus, Blood 2015; Merle, JCI-insight 2018]. We reported intravascular hemolysis and heme-loaded vesicles in common vascular conditions, like transfusion [Hariri, Critical Care 2019].

However, proof of concept that hemolysis occurs in chronic metabolic diseases, such as T2 diabetes, remained to be established.

Here we provide the first evidence that modifications of red blood cells -modifications that had been timidly reported in T2 diabetes- do result in low-grade, chronic, but significant intravascular hemolysis in diabetic patients.

We assessed several possibilities to quantify hemolysis in plasma, via red blood cell contents, or through their fragmented membranes. However, T2 diabetes imposes specific modifications onto red blood cells, making circulating vesicles impractical as biomarkers. Conversely, the measurement of cell-free heme in plasma, using simple but little-considered methods, helped demonstrate that *bona fide* intravascular hemolysis does occur in T2 diabetes. Plasma heme was specifically associated with peripheral neuropathy.

Extracellular vesicles from diabetic red blood cells were pathological heme carriers: They activated endothelial cells and promoted thrombin generation in plasma. This was successfully attenuated by heme-scavenging and PS-neutralizing proteins.

Our findings shed new light on the physiopathology of T2 diabetes, they add hemolysis as a substantial new pathway involved in the propagation of vascular inflammation. This data may help promote the consideration and development of novel therapeutic strategies to limit the effects of chronic intravascular hemolysis and vascular injury during T2 diabetes.

We declare that all authors concur with the submission and that the material submitted for publication has not been previously reported and is not under consideration for publication elsewhere.

**Response to reviewers**

First, we’d like to thank the reviewers for their very positive appreciation of the manuscript.

In the 1^st^ round of revision, the editors recommended some style modifications, some clarifications, and one of Reviewer #2 had a single request, for added discussion on a specific topic. All points were addressed and responses are provided below in extenso:

**Journal requirements**

**1**. **File naming**:

We ensured that the manuscript sticks more closely to the PLOS ONE style, and modified some of the section, figure and table titles accordingly, the captions and citing styles, throughout the document.

**2**. **Participant consent**:

We provide the mentioned additional details regarding participant consent. We checked that the phrase ‘written consent’ is indeed present in extenso in the text. We regrouped two sentences into one more definitive statement. Please, see **Page 4** in last paragraph.

**3**. **Data not shown**:

We have removed the phrase ‘data not shown’ from the manuscript. On one hand, we inserted actual data in text (**Page 12, top paragraph, as below**). On the other hand, we inserted a new table (**S3 Table**) gathering all the statistics linked to the absence of association of circulating levels of vesicles with vascular injury markers in T2D patients, to sh

ow the actual data. Please see Results, **Page 12**:

“Finally, circulating levels of PS+ and CD235a+ large EV were not statistically elevated in T2D, and large EV did not correlate with the circulating levels of HbA1c (R = -0.384; *p*>0.055 after adjustment for age and sex). PS+ and CD235a+ large EV were not associated with dyslipidemia, HTN or obesity as cardiovascular risk factors, nor with vascular damage in T2D patients (S3 Table).”

Please, see **S3 Table** in **Supporting Information**, and the matching legend **page 21**.

**4**. **Financial disclosure**:

We improved our financial disclosure statement by replacing our sentence « The funders had no bearing on the conduct of the study », with the phrase below:

« The funders had no role in study design, data collection and analysis, decision to publish, or preparation of the manuscript. »

**Funding included** Project ‘RépaDia’ of the Fondation de France to OBB, Project ‘BiFace’ of the Fondation pour la Recherche Médicale to OBB, Project ‘Cardannex’ of Inserm Transfert to OBB.

**5. Captions for your Supporting Information files**:

We improved the captions for Supporting Information. This includes titles of the different sections, and expanded captions for the 2 tables. Please, see **page 21** at end of manuscript.

**Submission:**

The Figure files were uploaded to the **PACE** digital diagnostic tool and seemed appropriate.

**Reviewer comments and requirements**

**Reviewer #2 quote:** [,,,] I have **only one concern**: in the methods the authors write the following: « We did not attempt to specify the different forms of ferrous, ferryl and deoxy-Hb which are all expected to contribute to Abs575. »

Pathological consequences of IVH are mainly due to the production and actions of oxidized Hb forms. I am missing the description of Hb oxidation, and the specific pro-oxidant and pro-inflammatory actions of the different products (metHb, ferrylHb, covalently crosslinked Hb forms, free heme). I understand that measuring the concentration of these products are quite challenging and it was not the goal of the current work, but this should be discussed properly to encourage further work in this field.

We addressed this concern by incrementing the discussion as suggested, beyond the notions already introduced. We inserted a new paragraph discussing more specifically the different forms of Hb oxidation products, and listing their specific effects on blood vessels. Please, see l3 last paragraphs of the discussion; **page 18** (bottom) and **19** (top), as well as the additional, recent reference by [**Bozza et al, Front Immunology, 2020**] necessary to support the description of Hb oxidation **(Reference #49 Bozza)**.

We also added a paragraph pointing at the future possibilities of quantifying the relative Hb degradation products specifically.

“ These products of Hb oxidation can stimulate specific pro-oxidant and pro-inflammatory actions on the vascular endothelium, with functional consequences. MetHb triggers the endothelial production of interleukin-6 and -8. FerrylHb can trigger endothelial activation especially through Nuclear Factor-kappa B (NFκB) pathway. Globin-derived radicals activate endothelial interleukin-1ß production via the NOD-like receptor family, pyrin domain containing 3 (NLRP3) inflammasome (49). In addition, oxidized forms of Hb shed their prosthetic heme more readily. Some suppose that protein-free heme could trigger specific oxidative reactions in the bloodstream, like hydroxyl radical formation through Fenton reaction or lipid peroxidation (49). However, opportunities for heme to maintain itself in a ‘free’ state are unlikely and would be extremely brief. The odds are that heme will rapidly be exchanged and interact with proteins present in plasma at high concentrations, like lipoproteins, hemopexin or albumin.

It would be advantageous to know which forms of oxidized Hb and derivatives prevail in T2D plasma in order to imagine therapeutic strategies for their neutralization. Future studies could attempt to distinguish Hb oxidation products in T2D, possibly via absorbance spectroscopy with spectral deconvolution techniques (26,50) or via reverse-phase high performance liquid chromatography (RP-HPLC) (34) and quantify their

relative proportions. ”

In conclusion, we are glad that you found our findings relevant, and hope that the new format and additions make the manuscript suitable for publication in PLOS One.

With our best regards,

Olivier Blanc-Brude, Ph.D.

Paris Cardiovascular Research Center, INSERM U970,

Hôpital Européen Georges Pompidou, centre de recherche,

56 rue Leblanc, 75015 Paris, France

Phone: 33-6-24-90-13-31 ; Email: [olivier.blanc-brude@inserm.fr](mailto:olivier.blanc-brude@inserm.fr)
